# Development of an instrument to assess social functioning in dementia: The Social Functioning in Dementia scale (SF-DEM)

**DOI:** 10.1016/j.dadm.2017.02.001

**Published:** 2017-02-24

**Authors:** Andrew Sommerlad, David Singleton, Rebecca Jones, Sube Banerjee, Gill Livingston

**Affiliations:** a Division of Psychiatry, University College London, London, UK; b Camden and Islington NHS Foundation Trust, St Pancras Hospital, London, UK; c Centre for Dementia Studies, Brighton and Sussex Medical School, University of Sussex, Brighton, East Sussex, UK

**Keywords:** Dementia, Social functioning, Assessment tool, Outcome assessment

## Abstract

**Introduction:**

Social functioning is a core domain in the life of people with dementia, but there is no accepted instrument to measure it. We aimed to develop the Social Functioning in Dementia (SF-DEM) scale and test its psychometric properties for assessing social function in people with dementia.

**Methods:**

We interviewed people with mild dementia and family caregivers to develop patient and caregiver-rated SF-DEM versions and refined them through interviews with health care professionals. We tested its psychometric properties in 30 dyads of people with dementia and family caregivers.

**Results:**

Both SF-DEM versions had content validity and demonstrated concurrent validity against a single item rating overall social functioning (patient rated *r* = 0.42, 95% CI [0.07–0.68]; caregiver rated *r* = 0.59, 95% CI [0.29–0.78]). All participants found it acceptable. Analyses showed reliability (test–retest, inter-rater, internal consistency) and indications of responsiveness to change.

**Discussion:**

SF-DEM shows promise as a valid, reliable, acceptable measure of social functioning in dementia.

## Highlights

• Social function is key to dementia diagnosis and important to patients and families.
• SF-DEM is the first assessment tool for measuring social functioning in dementia.
• In SF-DEM, core social functioning domains are assessed by self- or proxy-report.
• These measures are acceptable to clinicians and respondents, and are reliable.
• SF-DEM has content and concurrent validity in mild dementia.

## 1 Introduction

Dementia diagnostic criteria specify impairment, in activities of daily living or social function, must accompany cognitive decline [1], [2]. Changes in social function, “how individuals associate and interact, both in society at large and their own personal environment” [3], such as loss of interest in previously valued hobbies or changes within close relationships, are distressing to people with dementia [4], [5] and their families [6], [7], especially when the person with dementia lacks awareness of social changes [8]. Changes in social behavior occur in the early stages of a number of dementia subtypes [9] including Alzheimer's disease [10] and frontotemporal dementia [11]. These changes may be caused by emotion recognition [12] or theory of mind [13] deficits, or disinhibition [14] related to amygdala and frontal cortex network disruption [15]. Lower premorbid social functioning has been reported to increase dementia risk [16], [17], [18] and its progression [19]. Social function is therefore central to the diagnosis of dementia and is a core domain when considering etiology and progression and evaluating the effects of interventions in dementia.

Although measures of general function [20] and quality of life [21] include individual questions about social function, there is no validated instrument available to assess social functioning in people with dementia. We therefore aimed to develop a psychometrically sound and acceptable interviewer-administered measure of social functioning in dementia, the Social Functioning in Dementia (SF-DEM) scale, to be completed in a face-to-face interview with the person with dementia (self-report) or their family caregiver (proxy report).

## 2 Method

**Fig. 1:**
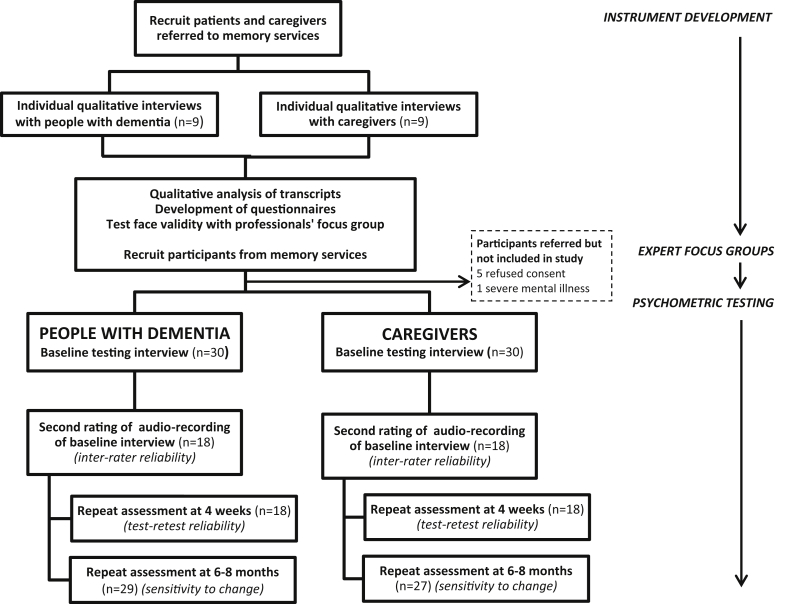
Overview of study design.

Westminster NRES Committee (15/LO/0105) gave ethical approval. We used gold-standard methodology [22] to develop and test the instrument in an iterative process (Fig. 1) in three phases: (1) instrument development—generation of domains and candidate questionnaire items through qualitative interviews with people with dementia and their family caregivers and a structured literature review; (2) expert interviews—qualitative interviews with dementia experts about the test structure and content to refine the draft assessment tools and test content validity; (3) psychometric testing—in structured interviews with people with dementia and their caregivers.

### 2.1 Instrument development

#### 2.1.1 Setting

We recruited participants from two community-based memory clinics in London, UK.

#### 2.1.2 Participants

We purposively sampled dyads of people with dementia and their family caregiver for a range of demographic and clinical characteristics to cover varied experiences of social changes in dementia. We stopped interviewing when no new content arose (theoretical saturation).

We included English speakers with dementia of any subtype (diagnosed clinically by consultant psychiatrists, then validated by A.S. against *Diagnostic and Statistical Manual of Mental Disorders, Fifth Edition* (DSM-V) criteria for major neurocognitive disorder [1]), of mild severity (Mini–Mental State Examination [MMSE] [23] score ≥20). We excluded those with severe physical or other mental illness limiting their participation in the interviews or those who lacked capacity to give informed consent.

We included current, English-speaking, unpaid, main caregivers, over 18 years old, in contact with a person with dementia at least weekly. We excluded caregivers with severe physical or mental illness which limited their participation in the interviews or without capacity to give informed consent.

#### 2.1.3 Procedure

A health care professional approached potential participants during clinical contact. Interested participants, after being provided with an information sheet, were approached for informed written consent. One researcher (A.S.) conducted the individual, audio-recorded, qualitative interviews, lasting 30–60 minutes, at participants' homes, with people with dementia and their caregivers separately to facilitate open discussion.

Our interview guide asked about the following:

• Important social activities and aspects of social relationships for person with dementia.
• Changes in the social relationships and activities of a person with dementia, the timing of changes, and their effects.
• Acceptability and ease of use of different question formats. We asked about formats from two current scales: one measuring quality of life in dementia (QOL-AD) including a question about social functioning, and the other a generic Social Functioning Scale [24], [25].

#### 2.1.4 Analysis

We transcribed recordings and checked transcriptions for accuracy. Two researchers (A.S. and D.S.) analyzed all transcripts to identify a conceptual framework of important social functions, guiding the selection of candidate items for SF-DEM.

### 2.2 Expert qualitative interviews

#### 2.2.1 Setting and participants

We purposively sampled health care professionals from participating memory clinics to achieve a range of characteristics: sex, experience in clinical and research settings, ethnicity, and specialty. We conducted one focus group and two individual interviews at their workplaces.

#### 2.2.2 Procedure

We developed an interview guide which

• tested content validity by inquiring whether our draft instruments balanced all important facets of social functioning and
• assessed clarity, acceptability, and ease of use.

### 2.3 Psychometric testing

#### 2.3.1 Setting and participants

The setting and inclusion and exclusion criteria for participants were the same as for the qualitative interviews. Consenting participants from those interviews took part in psychometric testing, and we recruited additional dyads.

#### 2.3.2 Procedures

A.S. conducted all semistructured interviews with people with dementia and their caregivers, at their homes at baseline; 4 weeks later to assess test–retest reliability; and 6–8 months later to assess responsiveness to change. Another researcher, D.S., completed SF-DEM for 18 dyads based on audio-recorded interviews, to assess interrater reliability between the two researchers.

#### 2.3.3 Measures

Baseline and 4-week interviews for people with dementia and caregivers included the following:

• Demographic characteristics: age, sex, ethnicity, marital status, education, employment status, and living arrangements (baseline only).
• SF-DEM instrument, our newly devised 20-item interviewer-administered instrument by self-report and proxy report.
• The question “How acceptable did you find this questionnaire?” with ratings either: Very acceptable; Acceptable; Unacceptable; Very unacceptable.

At baseline interviews for people with dementia:

• We chose the social domain question from Health Status Questionnaire (HSQ)-12 [26], a validated interview as there was neither a “gold-standard” test of social function, nor a social function measure for dementia against which to test our instrument's validity.
  - “During the past 4 weeks, to what extent has your memory problem interfered with your normal social activities with family, friends, neighbors, or groups?”

Rated as: Not at all; Slightly; Moderately; Quite a bit; or Extremely.

• MMSE [23].

At baseline interview for caregivers:

• We chose three social questions from a validated measure of quality of life, QOL-AD [24] to test our tools' construct validity.
  - “How is [your relative]'s relationship with their family members?”
  - “How is [your relative]'s relationship with the person closest to them [could be the caregiver]?”
  - “How is [your relative]'s relationship with their friends?”

Each rated as Poor; Fair; Good; or Excellent, and these ratings were scored as 1 to 4, generating a total score out of 12.

At 6- to 8-month follow-up interviews for people with dementia and caregivers:

• Changes to living arrangements or bereavement of patient or family caregiver
• SF-DEM instruments
• Three social questions from QOL-AD.
• MMSE (people with dementia only)

#### 2.3.4 Sample size calculation

Our a priori sample size calculation assumed nonparametrically distributed data and provided 80% power and a significance level of 5%. We calculated we would require 28 participants to find a correlation of >0.3 between our instrument and the social function domain of HSQ-12, assuming the true correlation to be 0.7. A sample size of 24 participants would detect a correlation of >0.7 between repeated interviews and 14 participants could detect correlation of >0.6 between raters, assuming the true correlation to be 0.9.

#### 2.3.5 Analysis

We compared results from patient-rated and caregiver-rated SF-DEM using paired t-tests. We assessed overall internal consistency using Cronbach's α and item-total and item-item reliability using Spearman's rank coefficient as this tested ordinal data. We tested interrater and test–retest reliability for total SF-DEM scores using the intraclass correlation coefficient (ICC) [27] using the (2,1) model for interrater reliability and (1,1) model [28] for test–retest reliability, and interrater and test–retest agreement for individual questions using Cohen's κ; we used quadratic weighting for κ as our ordinal rating scale had nonlinear ratings [29]. For validity testing, we used Spearman's rank coefficient to assess correlation of SF-DEM measures and ordinal data from HSQ-12 or QOL-AD questions and ICC for agreement between patient and caregiver. To test responsiveness to change, we used linear regression to assess the association between change in SF-DEM and an ordinal rating by the participant of the overall social change during the preceding year [30].

We used SPSS version 20 for all descriptive and analytical statistics apart from weighted-κ, which we calculated using Stata version 12.

## 3 Results

### 3.1 Instrument development

Table 1 shows the 18 participants' demographic and clinical characteristics. The mean age of the nine participants with dementia was 79 years (standard deviation [SD] 8) and five (56%) were male. The mean age of the nine caregivers was 68 years (SD 11) and two (22%) were male. We identified the following themes from our analysis and devised candidate items for the questionnaire based on the phrases our participants used and structured around the domains of our conceptual framework (for detail, see Appendix A).

**Table 1:** Clinical and demographic characteristics of participants in development and testing phase of SF-DEM social functioning instrument

Characteristic	Instrument development		Psychometric testing	
	Patient (*n* = 9)	Caregivers (*n* = 9)	Patient (*n* = 30)	Caregivers (*n* = 30)
	Mean (SD), range			
Age (years)	79 (8), 66–92	68 (11), 43–76	80 (8), 65–97	65 (13), 38–88

	*n* (%)			

Gender				
Female	4 (44)	7 (78)	15 (50)	24 (80)
Ethnicity				
White British	5 (56)	7 (78)	20 (67)	21 (70)
White other	3 (33)	1 (11)	7 (23)	6 (20)
Black and minority ethnic	1 (11)	1 (11)	3 (10)	3 (10)
Marital status				
Married	5 (56)	7 (78)	13 (43)	19 (63)
Single/common law	2 (22)	1 (11)	3 (10)	7 (23)
Widowed	1 (11)	1 (11)	9 (30)	1 (3)
Divorced/separated	1 (11)	0 (0)	5 (17)	3 (10)
Level of education				
Primary-school level	3 (33)	2 (22)	15 (50)	10 (33)
Secondary-school level	1 (11)	1 (11)	5 (17)	8 (27)
Post-secondary	5 (56)	6 (67)	10 (33)	11 (40)
Main occupation∗ (current or previous)				
Managerial or professional	5 (56)	5 (56)	10 (33)	10 (33)
Technical, clerical, or service	3 (33)	3 (33)	12 (40)	16 (53)
Craft or skilled labor	0 (0)	1 (11)	1 (3)	1 (3)
Machinery or elementary	1 (11)	0 (0)	7 (23)	2 (7)
Unemployed	0 (0)	0 (0)	0 (0)	1 (3)
Retired	8 (89)	7 (78)	27 (90)	16 (53)
Living situation of patient				
Lives alone	4 (44)		10 (33)	
Lives with spouse/partner	3 (33)		11 (37)	
Lives with other family	2 (22)		9 (30)	
Caregiver and patient living together	5 (56)		18 (60)	
Relationship to caregiver				
Spouse	5 (56)		15 (50)	
Parent/child	1 (11)		10 (33)	
Friend	2 (22)		2 (7)	
Other relation	1 (11)		3 (10)	
Dementia subtype†				
Alzheimer's disease	7 (78)		22 (74)	
Mixed (Alzheimer's/vascular)	1 (11)		5 (17)	
Parkinson's disease dementia	0 (0)		2 (7)	
Unspecified dementia	1 (11)		1 (3)	

	Mean (SD), range			

Patient's Mini–Mental State Examination score	27 (3), 20–29		26 (3), 20–30	

Abbreviations: SF-DEM, Social Functioning in Dementia; SD, standard deviation.

∗ Classified according to United Nations Statistics Division's *International Standard Classification of Occupations*.

† Dementia diagnosis validated against DSM-IV criteria, dementia subtype as recorded in clinical notes.

Participants told us about activities they had previously enjoyed, but where engagement had declined when participants developed dementia; including engaging socially with family and friends at home or at others' home and speaking to people on the phone, e-mail or social media, and hobbies enjoyed by people with dementia currently, such as going to cafés or social clubs. Caregivers told us about changes in the person with dementia which impeded social function, such as difficulty making or following conversation, especially in larger groups; increased critical comments; irritability; or loss of interest. People with dementia would frequently deny these changes or attribute them to age or physical ill health.

### 3.2 Professional experts

We interviewed three consultant psychiatrists, two psychologists, four nurses, and two support workers in a focus group or individual interviews. They commented on the range of activities covered in our instruments and suggested additional items and changes to the format of the instruments. We changed our instruments based on these interviews (Appendix A).

#### 3.2.1 Additional items

Professionals suggested adding summary questions asking whether the patient's social life was better or worse than 1 year before and whether they wanted to make changes to their social function. These could make the instruments a stimulus for conversation about past changes, and possible future lifestyle changes.

#### 3.2.2 Suggested changes to instrument format

The changes suggested were as follows:

• A shorter questionnaire to increase acceptability.
• Interviewer-administered questionnaire.
• Increase the time period covered from 2 to 4 weeks to permit a greater range of social activities.
• A prompt card for the four-item response options to aid understanding.
• An accompanying manual with detailed information on administering and scoring the instrument.

#### 3.2.3 Content validity

Participants told us the items in our instrument corresponded with the range of social activities and changes in social functioning that they see in people with dementia.

### 3.3 Format of SF-DEM instruments

The SF-DEM used in the testing phase (accessible at www.ucl.ac.uk/psychiatry/SFDEM) consists of 17 core questions scored, from 0 to 3, and three unscored summary questions. There are separate self- and caregiver-report forms using the same items but rephrased in the caregiver version for proxy response. Higher scores represent better social functioning; the maximum possible score is 51. In section 1, 11 questions cover engagement with social contacts and important social activities and, in section 2, 6 questions cover difficulties in social relationships and these are reverse scored.

### 3.4 Psychometric testing

Table 1 summarizes demographic and clinical characteristics of the 30 recruited dyads. People with dementia had a mean age of 80 years (SD 8) and half were women. The caregivers' mean age was 68 years (SD 13) and 80% were women. Participants came from a variety of ethnic backgrounds, educational levels, and previous employment. Half of dyads were spouses. Nearly three-quarters of participants had Alzheimer's disease.

#### 3.4.1 Descriptive statistics

Table 2 details the SF-DEM questions and participants' mean and range of scores. People with dementia used a full range of scores in nine questions and caregivers in 14 questions. The rating by people with dementia was higher than caregivers' (mean difference 4.1, 95% CI [1.9–6.2], *P* < .0005) because of difference in rating social relationship difficulties (section 2 mean difference 3.2, 95% CI [1.5–4.8], *P* < .0005). There was no significant difference between patient and caregiver rating for frequency of social contact and activity.

**Table 2:** Summary of participants' responses and scores on SF-DEM at baseline interview

SF-DEM domain: How often in the past month have you/they…	Patient rated (n = 30)				Caregiver rated (n = 30)			
	Frequency (%)				Frequency (%)			
	Very often	Often	Occasionally	Never	Very often	Often	Occasionally	Never
Social activities								
1. Seen friends or family in own home	22 (73)	8 (27)	0 (0)	0 (0)	21 (70)	7 (23)	2 (7)	0 (0)
2. Visited friends or family at their homes	2 (7)	9 (30)	9 (30)	10 (33)	1 (3)	10 (33)	5 (17)	14 (47)
3. Contacted friends or family by phone or computer	8 (27)	14 (47)	7 (23)	1 (3)	12 (40)	7 (23)	7 (23)	4 (13)
4. Attended community or religious meetings	0 (0)	5 (17)	4 (13)	21 (70)	0 (0)	6 (20)	3 (10)	21 (70)
5. Gone shopping with friends or family	0 (0)	16 (53)	4 (13)	10 (33)	1 (3)	15 (50)	3 (10)	11 (37)
6. Gone on trips or to events like the cinema or talks	0 (0)	11 (37)	7 (23)	12 (40)	0 (0)	6 (20)	10 (33)	14 (47)
7. Gone to a cafe, restaurant, pub, or social club	1 (3)	18 (60)	7 (23)	4 (13)	1 (3)	20 (67)	6 (20)	3 (10)
8. Exercised, walked, or played sport with others	0 (0)	8 (27)	3 (10)	19 (63)	1 (3)	7 (23)	4 (13)	18 (60)
9. Started or taken part in a conversation	25 (83)	4 (13)	1 (3)	0 (0)	20 (67)	7 (23)	2 (7)	1 (3)
10. Talked to others about your/their feelings or concerns	2 (7)	6 (20)	10 (33)	12 (40)	3 (10)	7 (23)	6 (20)	14 (47)
Personal relationships								
11. Asked other people about their feelings or concerns	0 (0)	11 (37)	6 (20)	13 (43)	2 (7)	3 (10)	6 (20)	19 (63)
12. Found it difficult to think of something to say to others	1 (3)	4 (13)	7 (23)	18 (60)	5 (17)	10 (33)	5 (17)	10 (33)
13. Found other people's conversation unclear	0 (0)	2 (7)	11 (37)	17 (57)	5 (17)	11 (37)	6 (20)	8 (27)
14. Been outspoken about what you/they really think	2 (7)	5 (17)	12 (40)	11 (37)	2 (7)	8 (27)	7 (23)	13 (43)
15. Found that other people are irritating	5 (17)	1 (3)	10 (33)	14 (47)	2 (7)	12 (40)	6 (20)	10 (33)
16. Had an argument or shouted at other people	3 (10)	2 (7)	6 (20)	19 (63)	1 (3)	3 (10)	10 (33)	16 (53)
17. Found they don't want to do things you/ they would usually	3 (10)	3 (10)	7 (23)	17 (57)	6 (20)	11 (37)	5 (17)	8 (27)

	Mean (SD)	Range			Mean (SD)	Range		
Summary scores								
Section 1 “Social activities” (1–11)	15.3 (3.7)	8, 23			14.3 (4.6)	6, 23		
Section 2 “Personal relationships” (12–17)	14.1 (3.3)	7, 18			10.9 (3.9)	3, 18		
Total	29.4 (5.4)	16, 38			25.3 (6.2)	16, 36		

	Patient rated (*n* = 30)				Caregiver rated (*n* = 30)			
	Frequency (%)				Frequency (%)			
	Excellent	Good	Fair	Poor	Excellent	Good	Fair	Poor
Thinking about your/their social life as a whole, how is it now?	6 (20)	13 (43)	7 (23)	4 (13)	3 (10)	10 (33)	12 (40)	5 (17)

	A lot better	A bit better	No change	A bit worse	A lot worse	A lot better	A bit better	No change	A bit worse	A lot worse
How is it now compared to 1 year ago?	3 (10)	5 (17)	15 (50)	5 (17)	2 (7)	2 (7)	3 (10)	15 (5)	8 (27)	2 (7)

	Rather do more	No change needed	Rather do less		Rather do more	No change needed	Rather do less	
Would you like your/their social life to change?	11 (37)	19 (63)	0 (0)		24 (80)	5 (17)	1 (3)	

Abbreviations: SF-DEM, Social Functioning in Dementia; SD, standard deviation.

NOTE. For each question, higher score indicates better social functioning. For questions 1–11 (section 1): 0 = never, 1 = occasionally, 2 = often, 3 = very often; for questions 12–17 (section 2): 0 = very often, 1 = often, 2 = occasionally, 3 = never.

#### 3.4.2 Acceptability and time burden

We first considered the presence of missing data and floor/ceiling effects for summary scores. There was 100% completion of all items in both forms, and no participants refused or were unable to complete the interview. There was no floor or ceiling effect as no one scored the minimum or maximum score on SF-DEM. All participants rated the instrument as acceptable (62%) or very acceptable (38%). Administration took a mean of 13 minutes (SD 5) for people with dementia and 11 minutes (SD 4) for caregivers.

#### 3.4.3 Internal consistency

Consistency for the patient-rated (α = 0.62, 95% CI [0.40–0.84]) and caregiver-rated instruments (α = 0.64, 95% CI [0.44–0.85]) was at an acceptable level for early-phase research [31]. Four items (4, 7, 8, 10) in the patient-rated instrument and two items (2, 15) in the caregiver-rated instrument had low item-total reliability (Table 3). We considered removing them but judged this would reduce the instruments' content validity. Item–item reliability was <0.5 between most items, although higher in four instances in each instrument (Appendix B). We considered whether any of these items could be eliminated but this would reduce the overall internal consistency. All items in SF-DEM were therefore retained.

**Table 3:** Summary of psychometric properties for individual items of SF-DEM

Psychometric property	Item-total correlation		Interrater reliability		Test–retest reliability		Convergent validity: patient–caregiver agreement
Statistic	Spearman's *r*		Cohen's quadratic-weighted κ		Cohen's quadratic-weighted κ		Cohen's quadratic-weighted κ
SF-DEM domain	Patient rated	Caregiver rated	Patient rated	Caregiver rated	Patient rated	Caregiver rated	
1. Seen friends or family in own home	0.25	−0.35	1.0	1.0	0.60	0.73	*0.36*
2. Visited friends or family at their homes	0.32	*0.52*	1.0	1.0	*0.33*	0.61	0.55
3. Contacted friends or family by phone or computer	0.23	0.60	0.95	1.0	*0.28*	0.44	0.52
4. Attended community or religious meetings	*0.08*	−0.04	1.0	1.0	0.74	0.78	0.76
5. Gone shopping with friends or family	0.34	0.07	1.0	1.0	0.55	0.75	0.63
6. Gone on trips or to events like the cinema or talks	0.20	0.32	0.96	1.0	*0.31*	0.68	0.69
7. Gone to a cafe, restaurant, pub, or social club	*−0.15*	0.11	1.0	0.96	*0.33*	0.95	0.54
8. Exercised, walked or played sport with others	*−0.07*	0.13	1.0	0.97	0.60	0.65	0.84
9. Started or taken part in a conversation	0.44	0.49	1.0	0.96	0.72	0.47	0.47
10. Talked to others about your/their feelings or concerns	*0.19*	0.25	1.0	1.0	0.54	*0.20*	*0.20*
11. Asked other people about their feelings or concerns	0.39	0.24	1.0	0.93	0.50	0.65	*0.12*
12. Found it difficult to think of something to say to others	0.23	0.38	1.0	1.0	0.93	0.72	*0.27*
13. Found other people's conversation unclear	0.22	0.34	1.0	1.0	*0.20*	0.56	*0.18*
14. Been outspoken about what you/they really think	0.20	0.03	*0.71*	1.0	*0.15*	0.67	*−0.19*
15. Found that other people are irritating	0.48	*0.22*	0.79	0.97	0.47	0.49	*0.14*
16. Had an argument or shouted at other people	0.32	0.16	0.78	1.0	*0.22*	*0.39*	*0.37*
17. Found they don't want to do things you/they would usually	0.42	0.57	0.79	1.0	*0.31*	0.82	*0.34*
Total	0.62	0.64	0.99	0.99	0.89	0.94	0.59
95% CI			0.99, 1.00	0.99, 1.00	0.70, 0.96	0.85, 0.98	0.07, 0.81
Statistic for total score	Cronbach's α		Intraclass correlation coefficient for total score				

Abbreviations: SF-DEM, Social Functioning in Dementia; CI, confidence interval.

NOTE. Statistics in italicized type indicate low reliability or agreement: item-total reliability: Cronbach's α would increase if item were deleted; interrater reliability: Cohen's quadratic-weighted κ ≤ 0.75; test–retest reliability: Cohen's quadratic-weighted κ ≤ 0.4; and correlation between patient and caregiver: Cohen's quadratic-weighted κ ≤ 0.4.

#### 3.4.4 Interrater reliability

Interrater correlation between the two researchers was very high for overall scores on the patient-rated SF-DEM (ICC = 0.99, 95% CI [0.99–1.00]) and the caregiver-rated SF-DEM (ICC = 0.99, 95% CI [0.99–1.00]) and exceeded suggested acceptability criteria [32]. Interrater agreement for individual items was good for one question and very good for all other questions (Table 3).

#### 3.4.5 Test–retest reliability

We repeated testing with SF-DEM after, on average, 29 days (SD 4, range 25–37) with 18 participants.

Test–retest correlation was very strong for the patient-rated (ICC = 0.80, 95% CI [0.54–0.92]) and caregiver-rated versions (ICC = 0.89, 95% CI [0.73–0.96]). Test–retest agreement for individual items was moderate or better (κ > 0.40) for 15 of 17 items on the caregiver-rated instrument and 9 of 17 items on the patient-rated instrument (Table 3).

#### 3.4.6 Validity

The assessment of validity is complicated by there being no psychometrically acceptable existing measure of social function against which to compare SF-DEM. Our prespecified construct validity test at baseline interview found no significant correlation between our patient-rated instrument and the social domain of HSQ-12 (*r* = −0.26, 95% CI [−0.57, 0.11], *P* = .17) and between our caregiver-rated instrument and the QOL-AD social domains (*r* = 0.33, 95% CI [−0.03, 0.62], *P* = .08). We added the patient-rated QOL-AD for our 6- to 8-month testing and found significant correlation between patient-rated QOL-AD and SF-DEM (*r* = 0.47, 95% CI [0.13, 0.72], *P* = .01) and between caregiver-rated QOL-AD and SF-DEM (*r* = 0.49, 95% CI [0.15, 0.73], *P* = .01).

There was significant moderate correlation of both SF-DEM measures with the summary SF-DEM question about overall impression of current social functioning (patient rated *r* = 0.44, 95% CI [0.07, 0.68], *P* = .01; caregiver rated *r* = 0.60, 95% CI [0.29, 0.78], *P* = .001). We assessed whether validity was affected by the patient's level of cognitive impairment, by dividing the cohort according to median MMSE score. Correlation between patient-rated SF-DEM total score and their summary impression of social functioning was very strong in the 18 participants whose MMSE ≥ 26 (*r* = 0.82, 95% CI [0.17, 0.83], *P* < .0005) but we found no significant correlation for the 12 participants with MMSE ≤ 25 (*r* = −0.44, 95% CI [−0.79, 0.24], *P* = .15).

We found moderate correlation (*r* = 0.59, 95% CI [0.07, 0.81], *P* = .001) between overall scores from our patient-rated and caregiver-rated instruments indicating convergent validity (mean agreement for section 1 κ = 0.52, section 2 κ = 0.19).

#### 3.4.7 Responsiveness

We repeated testing with SF-DEM, on average, 7.2 months (SD 15, range 182–251 days) later with 29 patients and 27 caregivers (Table 4). Patients' mean MMSE score reduced by 0.3 points (SD 2.5, range −6 to +5) and only one participant had experienced a major change in the intervening period; a family member had moved into their home.

**Table 4:** Responsiveness to change during 6- to 8-month follow-up period: Association of SF-DEM total score and overall impression of change

	SF-DEM total change, mean change (SD)	How is it now compared to one year ago? *n* (%)					B	SE	*P*
		A lot worse	A bit worse	No change	A bit better	A lot better			
Patient rated	−1.2 (3.1)	0	7 (24)	17 (59)	4 (14)	1 (3)	1.39	0.72	.06
Caregiver rated	0.1 (3.9)	4 (15)	7 (26)	14 (52)	2 (7)	0	1.33	0.79	.10

Abbreviations: SF-DEM, Social Functioning in Dementia; SD, standard deviation; SE, standard error.

We found a range of SF-DEM change. The mean patient-rated SF-DEM score decreased by 1.2 points (SD 3.1, range from 6 to −7), whereas the caregiver-rated SF-DEM increased by 0.1 points on average (SD 3.9, range from 12 to −8). For details of individual SF-DEM domains' change, see Appendix C. We found preliminary evidence for the participants' overall impression of social change during the past year predicting SF-DEM score. Patient-rated SF-DEM score increased by 1.3 points (95% CI [−0.3, 2.9], *P* = .10) and caregiver-rated SF-DEM score increased by 1.4 points (95% CI [−0.1, 2.9], *P* = .06) for each point on the five-point ordinal scale of social change. Change in MMSE score for the person with dementia was not associated with SF-DEM change; one-point decline on MMSE during the follow-up period was associated with a 0.24-point improvement on the patient-rated scale (*P* = .20) and 0.04 decline on the caregiver-rated scale (*P* = .84).

## 4 Discussion

We have developed the SF-DEM, which measures social functioning of people with dementia, based on interview with them and/or their family caregiver. Both self- and caregiver-report instruments are acceptable, internally consistent and have interrater and test–retest reliability and content, concurrent and convergent validity in people with mild dementia.

Total SF-DEM score and overall impression of current “social life” were rated higher by people with dementia than caregivers (63% of patients vs. 43% of caregivers rated social function as good or excellent). This difference was accounted for by items which required judgment and abstract understanding (section 2, cognitive and psychological barriers to social engagement). Lack of insight is common in dementia [33] and increases as the disease develops [34]. People with dementia rate their quality of life higher than observers [35] possibly partly because they underestimate social changes and the cognitive deficits underlying these changes. However, this fits with the “disability paradox” [36] where differences may be a true reflection of different appraisal by the patient and caregiver, rather than a function of error due to cognitive impairment. This underlines the additional value of caregiver rating and need for further psychometric evaluation in more severe dementia.

Testing the SF-DEM's validity is challenging as there is no psychometrically acceptable existing measure of social function with which to compare this new instrument and researchers cannot feasibly directly measure participants' social function (although families can). There was significant correlation between the SF-DEM and a summary item and social function questions from the QOL-AD. We found no correlation for patients when compared to a question from the HSQ-12, a general social functioning measure, possibly because it required social difficulties to be attributed to memory deficits, and therefore needed insight into cognitive and social changes. We found statistically significant moderate correlation for both patients and caregivers at follow-up, reflecting the instruments' construct validity. The moderate correlation between the patient- or caregiver-rated overall score of SF-DEM and their overall impression of the person's “social life” indicates SF-DEM's concurrent validity, and the agreement between patient and caregiver ratings supports the validity of our instrument. We did not find any association in this small sample between change in MMSE score and change in social functioning as measured by SF-DEM, which may reflect that multiple different factors, rather than the progression of cognitive impairment, contribute to change in social functioning in people with mild dementia. This lack of association with increasing cognitive impairment is also seen in health-related quality of life [37], another multidimensional construct. In addition, there was only a decrease of 0.3 in the mean MMSE score in this study so we had very limited power to detect change.

### 4.1 Strengths and limitations

The main strength of this study is our use of mixed-methodology, meeting quality criteria [38] for the development and testing of SF-DEM. We powered the study for baseline validation but not for ability to detect change. Study participants covered a range of inner city and suburban-dwelling older people with dementia who had contact with memory services and included participants from different settings, ethnic, and social backgrounds. Further work is needed to evaluate SF-DEM's psychometric properties in other populations, such as rural areas, and other cultures where there may be different influences on social function.

One limitation of this study is the restriction of the instrument's development to those with mild dementia which limits the current use of the SF-DEM to this group. Further work is needed to test SF-DEM's psychometric performance in those with moderate and severe dementia, to determine if it accurately measures social function later in the disease process, and also in a cognitively healthy older population, to assess whether early social changes are accurately identified. Another limitation is the relatively small size of the test population meaning that evaluation of the effect of sociodemographic and clinical characteristics on performance on the SF-DEM was not possible. The sample size also precluded factor analysis. The use of the SF-DEM in larger populations would enable the investigation of the effects of variables such as subtype of dementia; insight; affected cognitive domains; premorbid personality; comorbid physical and mental illness; and external factors such as level of support, caregiver coping, and home and neighborhood environment and opportunities.

All our interviews were completed by one researcher, increasing the consistency of questioning style. The psychometric properties may be less good when used by a number of interviewers, although the manual aims to standardize use. We tested interrater reliability, but our very high ratings were based on the second researcher listening to the assessments' audio recordings so would have been affected by the first rater's questioning style.

### 4.2 Clinical implications and further research

Our findings provide encouraging evidence that self- and caregiver-report SF-DEM can be used to measure social function in those with mild dementia. Further research is needed to test the instrument in those with moderate and severe dementia, to test its responsiveness to change more fully, and to assess its use in differing settings. Research in an independent multicenter UK-based clinical sample and other English-speaking countries would test the generalizability of our findings and establish normative data. A larger sample would allow factor analysis to further investigate SF-DEM's internal structure and guide possible refinement of the instruments.

Changes in social function in dementia, such as loss of previously valued social activities, and changes to social relationships are distressing for patients and their caregivers but may often not be assessed systematically in clinical settings. Using SF-DEM in clinical settings could facilitate awareness and conversation about distressing social changes, potentially supporting patients and their caregivers to make changes to improve social function. Detection of early dementia markers is a priority area in dementia research, and there is increasing interest in the concept of mild behavioral impairment, in which social changes are viewed as prodromal dementia symptoms [39]. Testing social function using SF-DEM may enable the detection of such changes.

**Research in Context**

1. Systematic review: The authors reviewed the literature using traditional (e.g., PubMed) sources. Despite social function being a core domain for dementia diagnosis and important to patients and their families, there is no validated measure of social functioning in dementia. Some measures of quality of life and activities of daily living in dementia ask individual questions about social function, and these relevant citations are appropriately cited.
2. Interpretation: We developed the first measure of social functioning in dementia (SF-DEM) and tested its psychometric properties in people with mild dementia. We found SF-DEM to be acceptable, reliable, and valid.
3. Future directions: SF-DEM will allow research into the determinants and mediators of social change in dementia and the impact of psychosocial and pharmacological interventions on patients' social function. Further research is required to test its responsiveness to change and generalizability to other populations. These measures are now freely available to other researchers.

## Supplementary data

Appendices A–C
