# Supplementary material for: Development of an instrument to assess social functioning in dementia: The Social Functioning in Dementia scale (SF-DEM)
Source: Alzheimers Dement (Amst). 2017 Feb 24;7:88–98. doi: 10.1016/j.dadm.2017.02.001 (PMC5344217; doi:10.1016/j.dadm.2017.02.001)
Supplement: Appendices A–C [file mmc1.pdf]

Supplemental files - **Development of an instrument to assess social functioning in dementia: The Social Functioning in Dementia scale (SF-DEM)**

Appendix A: Themes from qualitative interviews with people with dementia and carers and focus groups with clinicians for development and testing of SF-DEM

	Category	Theme	Exemplar quote
Interviews with people with dementia and carers Framework for social functioning in dementia	Engagement with social contacts	Withdrawing from contact with friends or family	"I didn't go out for months and months … and I gave up all my friends … I didn't want them to know that I wasn't able to keep up." *P1,70 year old woman with dementia*<br>"Certainly when we were away on a group holiday, he was very withdrawn with other people; not with me but with other people." *C2,76 year old sister-in-law of man with dementia*
		Less likely to visit / call others	"She used to go [to visit her partner] but now they have a phone relationship." *C1, 43 year old daughter of woman with dementia*
	Important social activities	Involvement in church community	"I go to church every Sunday … I am also a trustee for the church." *P3, 85 year old man with dementia*
		Attending local café or restaurant	"We have agreed to go, but sometimes closer to the time, he sort of makes excuses as to why he doesn't want to go." *C4, 56 year old wife of man with dementia*
		Day trips, cinema or concerts	"He used to be more spontaneous: 'Let's go to the theatre or a concert'." *C4, 56 year old wife of man with dementia*
		Visiting family or friends	"I've been over to [neighbour]'s a couple of times in the last couple of weeks; just go in and have a chat and catch up with things." *P5, 66 year old woman with dementia*
		Shopping with family or friends	"She used to go shopping with me, but we don't do that any more." *C6, 71 year old husband of woman with dementia*
	Difficulties in social relationships	Less emotionally connected with others	"She never says 'are you alright darling?'." *C6, 71 year old husband of woman with dementia*
		Struggles to follow conversations	"I think sometimes it's easier not to hear the conversation or to pretend you don't hear the conversation so you don't have to engage in it." *C4, 56 year old wife of man with dementia*
		More forthright opinions	"I hear him say things that are very critical." *C7, 72 year old partner of man with dementia*
		More irritable or impatient with family or friends	"She'll ask you how to do something and you'll start to tell her and then she'll start arguing with you." *C8, 73 year old friend of woman with dementia*
		Embarrassed about people recognising their cognitive deficits	"If he started forgetting things, they would say 'oh, he's getting dementia' and he'd hide away again … he wouldn't want to go out." *C9, 73 year old wife of man with dementia*
		Reduced repertoire of conversation	"[Forgetting] makes my conversation with other people get less and less, you know, because I can't talk about things with them." P*2, 92 year old man with dementia*<br>"He can't really take in what's written in the papers or television … so that's not much good when it comes to conversation." *C2, 76 year old sister-in-law of man with dementia*
	**Category**	**Suggested change**	**Exemplar quote**
Interviews with experts	Additional items	Ask whether participants think there has been change in social function	
		Ask participants whether they would like to change their social function	"Just add the question 'would you like to do more'." *S1, Consultant psychiatrist in memory service*<br>"I think you need to add that question, because sometimes people say they are fine and don't want to change. We do so many assessment tools and we're not really sure if it is benefitting people." S2, *Nurse working in memory service.*
	Changes to instrument format	Abbreviate instrument to increase acceptability	"Maybe 10 or 15 minutes would be appropriate; and the more you do it the quicker you would get with asking these questions." S3, *Nurse working in memory service.*
		Simplify instrument completion and scoring	"Anything that makes it easier to go through, and it's done." S2, *Nurse working in memory service.*
		Define response categories	"You should explain what it means to say 'often' or 'occasionally', so you can clarify what they mean." *S4, Nurse working in memory service*
		Provide prompts for responses	"I think they could get more confused if they didn't have a prompt." *S5, Clinical support worker in memory service*

Supplemental files - **Development of an instrument to assess social functioning in dementia: The Social Functioning in Dementia scale (SF-DEM)**

Appendix B[1]. Item-item correlation matrix of individual questions from patient-rated SF-DEM instrument

		**SF-DEM domain**																
		**1**	**2**	**3**	**4**	**5**	**6**	**7**	**8**	**9**	**10**	**11**	**12**	**13**	**14**	**15**	**16**	**17**
**SF-DEM domain**	**1**																	
	**2**	-0.10																
	**3**	-0.31	0.27															
	**4**	0.17	0.03	0.08														
	**5**	0.38	0.17	0.01	-0.04													
	**6**	0.24	0.33	0.05	-0.28	-0.03												
	**7**	-0.07	0.16	0.14	-0.31	0.13	0.03											
	**8**	0.09	-0.20	-0.11	-0.24	0.22	0.03	0.14										
	**9**	0.38	0.19	0.16	-0.11	**0.55**	0.30	-0.35	0.14									
	**10**	0.12	0.20	0.27	0.19	-0.02	0.37	-0.33	-0.03	0.27								
	**11**	0.29	0.25	0.18	-0.05	0.14	0.38	0.00	0.10	0.20	0.44							
	**12**	-0.07	0.16	0.22	0.28	-0.02	-0.25	-0.33	-0.39	0.12	-0.05	0.12						
	**13**	-0.12	0.20	0.03	0.07	0.12	-0.03	-0.49	-0.15	0.34	-0.12	-0.12	**0.64**					
	**14**	-0.12	0.15	0.19	-0.24	0.15	0.09	0.05	0.33	0.35	-0.12	0.17	0.10	0.36				
	**15**	0.13	0.15	0.32	-0.02	0.39	0.04	-0.19	0.15	0.49	0.14	0.25	0.32	0.48	0.34			
	**16**	-0.01	0.07	0.14	-0.14	0.37	0.25	-0.22	-0.04	0.51	0.03	0.26	0.27	0.41	0.47	**0.50**		
	**17**	0.24	0.33	0.05	0.19	0.27	0.24	-0.41	-0.12	**0.60**	0.24	0.09	0.47	**0.59**	0.28	0.44	0.31	

Key: All statistics are r. Items with agreement ≥ 0.5 are in bold type.

Supplemental files - **Development of an instrument to assess social functioning in dementia: The Social Functioning in Dementia scale (SF-DEM)**

Appendix B[2]. Item-item correlation matrix of individual questions from carer-rated SF-DEM instrument

		**SF-DEM domain**																
		**1**	**2**	**3**	**4**	**5**	**6**	**7**	**8**	**9**	**10**	**11**	**12**	**13**	**14**	**15**	**16**	**17**
**SF-DEM domain**	**1**																	
	**2**	-0.50																
	**3**	-0.36	0.77															
	**4**	0.17	-0.04	0.17														
	**5**	0.07	-0.02	-0.08	-0.17													
	**6**	-0.07	0.33	0.17	-0.21	-0.11												
	**7**	0.08	0.16	0.08	0.03	0.06	0.31											
	**8**	-0.08	0.09	-0.03	0.02	0.28	0.30	0.09										
	**9**	-0.01	0.41	**0.59**	-0.05	0.17	0.07	-0.13	0.18									
	**10**	-0.07	0.36	0.29	-0.02	0.23	0.28	0.16	0.40	0.27								
	**11**	-0.20	0.12	0.13	0.14	0.31	0.04	0.19	0.40	0.07	**0.58**							
	**12**	-0.18	0.23	0.36	0.11	-0.03	-0.03	-0.20	-0.19	0.45	-0.15	-0.07						
	**13**	-0.35	0.20	0.34	0.14	-0.08	-0.06	-0.26	-0.10	0.45	-0.13	-0.11	**0.79**					
	**14**	-0.20	0.00	-0.03	-0.32	-0.08	0.32	0.16	-0.06	-0.29	-0.03	-0.13	0.04	-0.05				
	**15**	-0.12	0.09	0.27	-0.21	-0.06	0.11	-0.11	-0.17	0.19	-0.40	-0.24	0.40	0.34	0.42			
	**16**	0.07	-0.01	0.20	-0.13	-0.05	0.05	-0.06	-0.34	0.01	-0.43	-0.21	0.29	0.19	0.37	**0.69**		
	**17**	-0.33	0.54	0.41	0.10	0.03	0.40	0.20	0.05	0.28	0.22	0.33	0.25	0.39	0.05	0.06	0.21	

Key: All statistics are r. Items with agreement ≥ 0.5 are in bold type.

Supplemental files - **Development of an instrument to assess social functioning in dementia: The Social Functioning in Dementia scale (SF-DEM)**

Appendix C. SF-DEM changes from baseline assessment to 7 month follow-up

**Patient-rated / carer-rated SF-DEM**		**Section of SF-DEM**			**SF-DEM Domain**		
	Mean change (s.d.) (Range)		Mean change	(s.d.)		Mean change	(s.d.)
**Patient-Rated (n=29)**	-1.2 (3.1) (-7 to +6)	**Section 1**	-1.4	(2.4)	**1**	0.0	(0.3)
					**2**	-0.2	(0.9)
					**3**	-0.4	(0.8)
					**4**	0.1	(0.7)
					**5**	-0.1	(0.8)
					**6**	-0.3	(1.0)
					**7**	-0.2	(1.0)
					**8**	-0.2	(0.8)
					**9**	-0.1	(0.9)
					**10**	0.0	(1.0)
					**11**	0.1	(1.0)
		**Section 2**	0.2	(2.2)	**12**	0.0	(0.6)
					**13**	-0.1	(0.9)
					**14**	0.1	(0.8)
					**15**	-0.1	(0.8)
					**16**	0.2	(0.8)
					**17**	0.1	(0.8)
**Carer-Rated (n=27)**	0.1 (3.9) (-8 to +12)	**Section 1**	0.0	(2.5)	**1**	0.1	(0.5)
					**2**	-0.1	(0.9)
					**3**	-0.1	(0.9)
					**4**	0.1	(0.4)
					**5**	-0.1	(0.6)
					**6**	0.1	(0.9)
					**7**	-0.4	(0.7)
					**8**	-0.1	(0.8)
					**9**	0.1	(1.0)
					**10**	0.2	(0.9)
					**11**	0.2	(1.2)
		**Section 2**	0.1	(2.5)	**12**	-0.1	(1.1)
					**13**	0.3	(1.1)
					**14**	0.0	(0.9)
					**15**	-0.1	(0.9)
					**16**	0.1	(0.9)
					**17**	-0.1	(1.0)
